# Education Research: Quality of Narrative Feedback Generated by a Large Language Model Compared With Expert Faculty for Case-Based Learning in Neurology Education

**DOI:** 10.1212/NE9.0000000000200320

**Published:** 2026-05-27

**Authors:** Hannah Fruitman, Sasha Severin, Atikul Miah, Christina Gao, Haelynn Gim, Carolyn Qian, Sang-O Park, Kelly Hou, Edward L. Kong, Benjamin Cook, Jasmin Le, Brandon Stretton, John Maddison, Liam G. McCoy, Luke Collins, Andrew Vanlint, Rudy Goh, Matthew Arnold, Aye Thant, Rani Priyanka Vasireddy, Doris Kung, Ashley M. Paul, Haatem Reda, Tamara B. Kaplan, Adam Karp, Galina Gheihman

**Affiliations:** 1 New York Medical College, Valhalla, NY;; 2 Adelaide Medical School, The University of Adelaide, South Australia, Australia;; 3 Harvard Medical School, Harvard University, Boston, MA;; 4 Harvard/MIT MD-PhD Program, Boston, MA;; 5 Lyell McEwin Hospital, Elizabeth Vale, South Australia, Australia;; 6 Division of Neurology, Faculty of Medicine and Dentistry, University of Alberta, Edmonton, Canada;; 7 Massachusetts Institute of Technology, Boston;; 8 Washington University in St. Louis, School of Medicine, MO;; 9 University of Texas at Tyler School of Medicine;; 10 Baylor College of Medicine, Houston, TX;; 11 Johns Hopkins University School of Medicine, Johns Hopkins University, Baltimore, MD;; 12 Department of Neurology, Mass General Brigham, Boston, MA; and; 13 Department of Neurology, Westchester Medical Center, Valhalla, NY.

## Abstract

**Background and Objectives:**

Neurology learners often receive limited feedback in clinical settings because of workflow constraints, variability in supervision, and competing clinical demands. Artificial intelligence, including large language models (LLMs) may help address these gaps and provide clinical learners with effective formative feedback by generating real-time, case-specific feedback during neurology case-based learning (CBL). The aim of this study was to examine how the quality of LLM-generated feedback compares with human expert-generated feedback in neurology CBL.

**Methods:**

In this exploratory quantitative study, student participants undertook LLM-enabled interactive cases on the TEACHABLE platform, which included history gathering, physical examination elements, and ordering diagnostic testing. Participants were clinical-level students recruited from 2 medical institutions. Case transcripts were recorded and analyzed for feedback generation, which was provided by an LLM and human experts in 2 components: history taking/physical examination elements (H&P) and assessment and plan (A&P). Feedback characteristics including sentence count, word count, and reference to case key learning points were summarized and compared. Feedback quality was scored by blinded experts using the QuAL and EFeCT instruments. Results were compared for the H&P and A&P components of the case interactions.

**Results:**

Four student participants completed 5 interactive cases each, generating 20 total transcripts for feedback. Word and sentence number were similar among LLM-generated and expert-generated feedback, except for a greater word length in expert-generated A&P feedback. Regarding H&P, the LLM commented on the key learning points in 20/20 (100%) of the cases as compared with 39/60 (65%) for the human experts. For A&P, the LLM feedback discussed key points in 20/20 (100%) cases as compared with 39/40 (97.5%) for the human experts. The LLM feedback had no medical inaccuracies. QuAL and EFeCT scores were significantly greater for the LLM as compared with human experts for the H&P component, but not significantly different for the A&P component.

**Discussion:**

LLMs provided with key learning points can generate timely, quality feedback on case-based interactions in a manner comparable with human experts. A hybrid framework combining LLM-generated feedback with faculty input may offer high-quality and equitably accessible formative feedback at scale. These pilot findings are limited by a small sample size and experimental setting.

## Introduction

Artificial intelligence (AI) is reshaping the landscape of medical education. The efficiency and accessibility of large language models (LLMs) has facilitated their use by students across various fields, influencing open-book examinations,^1^ assessment preparation,^2^ and student information-seeking patterns.^3^ The use of AI-supported learning has the potential to bridge gaps in training by supplementing clinical experiences with diverse clinical scenarios and providing on-demand self-directed case-based learning (CBL).^4^ As these platforms continue to evolve, it is crucial to critically evaluate their effect and optimize their integration into medical education.^5^

One aspect of medical education in which AI integration may be useful is the development of clinical reasoning, a complex, multilayered skill that requires years of experience to master.^6^ Clinical reasoning can be refined through *deliberate practice* with clinical cases, a framework involving repetitive engagement in activities designed by educators, with specific objectives; measurable performance indicators; and effective, real-time feedback, which supports iterative improvement.^7^ Given that obtaining sufficient repetitions of practice in the clinical setting may be challenging, LLMs may help bridge this gap by offering opportunities for simulated practice. In pilot studies, we have shown that a gen-AI‑enabled LLM teaching platform, Transforming Education And Clinical Healthcare through Agent-Based Learning and Evaluation (TEACHABLE), can reliably, accurately, and effectively deliver clinical case simulations to learners with high fidelity.^8^ On this platform, hallucination rates were <1%^9^ and students engaged with originally written cases as well as those converted from published educational case reports.^10^

To make practice *deliberate*, however, there must be an element of timely feedback by an expert. Although feedback is critical in medical education, feedback opportunities for medical students in the classroom or clinical setting are often infrequent and inadequate.^11^ Barriers include time constraints, environmental aspects, or relationships among students and supervisors.^12^ Other barriers, including lack of faculty training in feedback skills and variable receptivity of students to feedback, may also play a role and affect feedback efficacy.^13,14^ When delivered effectively, feedback serves as more than performance evaluation—it is a key driver of learning.^15^

A promise of generative AI, and specifically LLMs, is the ability to generate timely, specific, and accessible written feedback for learners in a time-efficient manner at scale. LLMs can offer immediate feedback, are available at all hours (and in a learner-driven manner), and may standardize the consistency and reliability of feedback.^16,17^ What remains to be determined is whether AI-generated feedback is accurate and sufficient in quality as compared with faculty-generated feedback. The quality of feedback generated by an LLM (or by faculty) matters for formative assessment and guiding student improvement—–several instruments have been developed to assess quality of feedback in the medical setting and emphasize the feedback being learner-centered, specific, aligned with learning objectives, and actionable.^18,19^

The aim of this pilot study was to explore whether timely, appropriate LLM-generated narrative feedback could be delivered to learners on an AI-enabled CBL platform (TEACHABLE). We hypothesized that the LLM would deliver timely, appropriate narrative feedback to learners in an interactive, stepwise clinical reasoning environment and that it would do so with at least equal quality compared with feedback generated by human experts as assessed using 2 instruments previously validated for use in medical education settings.^18,19^

## Methods

### Study Design

This was an exploratory quantitative study with a positivist approach that compared the timeliness and quality of LLM-generated vs human expert‑generated feedback on student CBL as measured by rated scores on 2 quality-of-feedback instruments.

### AI-Enabled Learning Platform

**Figure 1:**
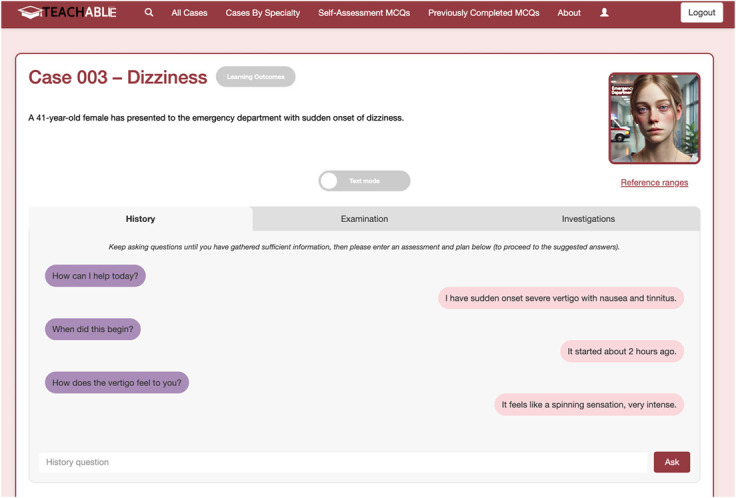
TEACHABLE Case Sample screenshot from the online platform TEACHABLE where students completed the case interactions.

We used an interactive, LLM-enabled CBL platform called TEACHABLE. This platform is a secure, freely accessible, web-based instructional environment that allows interactive dialog between a learner and an embedded LLM interface, permitting stepwise clinical reasoning within a standardized framework. Learners can use the platform to obtain history, request examination findings and investigation results, and formulate an A&P (Figure 1). In pilot studies in an experimental setting, we found TEACHABLE can deliver history, examination, and investigation content with negligible hallucinations in both neurology and perioperative medicine settings.^8,9^ The LLM interface is programmed to deliver automatic numerical and narrative feedback according to key points established by case authors. We found AI numerical scoring correlated with expert scoring; this study examined narrative feedback quality.^10^

### Case Selection and Review

TEACHABLE hosts a digital library of over 100 interactive clinical cases, written and peer reviewed by faculty. Cases represent diverse medical specialties, levels of complexity, and geographies. For this pilot study, 5 neurology cases were selected. All cases were reviewed previously by content experts to ensure reliable presentation by the LLM software and used in prior pilot testing to establish their validity for CBL. Cases were chosen because they represented common localization problems in neurology and required integration of history, examination, and investigation findings—skills that test foundational knowledge and clinical reasoning. Neurology was selected as the initial specialty for this pilot study because case-based reasoning in this field requires anatomical localization, which we believed would provide a clear structure for assessing learner diagnostic reasoning, and thus to evaluate the ability of an LLM to generate feedback related to clinical reasoning as compared with faculty experts. These neurology cases emphasized diagnostic reasoning over procedural skills, allowing focused analysis of feedback quality for cognitive learning. Each case underwent iterative pilot testing and review by neurologists, medical educators, and learners to ensure clarity, appropriate complexity, and relevant key learning objectives. A consensus among experts was reached regarding key points that should be elicited in the evaluation of each case, which became the learning points for which feedback was provided. The key case learning points are summarized in the Table and also provided in eAppendix 1 (history/physical examination) and eAppendix 2 (assessment and plan).

**Table:** Key Learning Points for Each of the Five Cases as Written by Faculty Case Authors for the History and Physical Examination Portion and Assessment and Plan Portion

Case title/topic	History and physical examination (H&P) key points	Assessment and plan (A&P) key points
1– Headache	1. Worst-ever headache 2. Not like usual migraines 3. Fever (38.4) 4. Limited range of motion of the neck (nuchal rigidity) 5. CRP elevated (119) 6. Lactate elevated (2.3) 7. CSF abnormal (high white blood cell count—pleocytosis—and high protein)	1. Meningitis 2. Bacterial 3. Antibiotics (such as ceftriaxone) 4. Dexamethasone 5. Chase CSF microbiology (such as cultures) 6. Admit
2– Upper limb weakness	1. Arm normal when went to bed 2. Alcohol consumption yesterday before bed 3. Weakness of wrist extension (distal) 4. Reduced (absent) reflexes over triceps and brachioradialis 5. Reduced sensation over the dorsal aspect of the hand	1. Radial neuropathy 2. Above level of spiral groove 3. Physiotherapy 4. Splint 5. Reduce alcohol consumption
3– Dizziness	1. Previous similar episodes of vertigo 2. 30 min in duration 3. Aural fullness (feeling of the ear being full) 4. Tinnitus 5. No incoordination or past pointing or ataxia	1. Probable Meniere disease 2. Antiemetics (such as ondansetron) 3. Intravenous normal saline 4. Consider betahistine 5. Trial dietary modification (reduce salt, caffeine, alcohol, and MSG) 6. Audiometry monitoring
4– Vision loss	1. Sudden onset (abrupt) vision loss 2. Headaches 3. Shoulder and hip girdle aching 4. Right-eye reduced visual acuity (hand movements) 5. Right RAPD (relative afferent pupillary defect) 6. Right retina has pale appearance, with a bright red spot 7. Brain CT with angiogram has no large vessel stenoses 8. ESR elevated 67 or CRP elevated 41	1. Likely central retinal artery occlusion (CRAO) 2. Giant cell arteritis (GCA) 3. Intravenous methylprednisolone 4. Temporal artery biopsy 5. Preimmunosuppression blood tests (e.g., HIV, hepatitis B, hepatitis C) 6. Admit
5– Diplopia	1. Weakness involving eyes, proximal arms and legs, and mouth 2. 6 mo in duration 3. Evidence of fatigability (such as worsens with chewing tough food or worst at the end of the day) 4. No impending respiratory failure (normal single breath count test and/or FVC results) 5. Myasthenic signs on examination (fatigable ophthalmoplegia or ptosis, or ice-pack test, or Cogan lid twitch) 6. Acetylcholine receptor antibodies (AChR) positive 7. Chest CT demonstrates anterior mediastinal mass (likely thymoma)	1. Myasthenia gravis 2. Not requiring ICU at this stage 3. Admission 4. FVCs and/or single breath count test (respiratory monitoring) 5. Blood tests for preimmunosuppression serology 6. IVIg or PLEX

### Student Participants

Four clinical-year medical student coinvestigators were recruited, 2 from Harvard Medical School in the United States and 2 from Adelaide University School of Medicine in Australia. Students interacted with patients with the aim of reaching a final A&P. Students obtained history, asked for examination findings, and requested investigations through a dialog box (Figure 1),^20^ then entered a final A&P. All student questions and LLM answers were automatically recorded in a transcript of their interaction by the TEACHABLE platform. After each simulated encounter, full anonymized transcripts were exported for feedback generation.

### Feedback Generation

We had 2 parts to our pilot study. First, we looked at LLM-generated and human-generated feedback focused on student performance of H&P elements. Second, we evaluated LLM-generated and human-generated feedback focused on student performance for A&P formulation for each case.

#### Part 1: History and Physical Examination Elements

For H&P, 3 experts (2 clinicians and 1 medical educator) reviewed each student transcript and provided narrative feedback in a free-text unstructured manner. The 2 clinicians were neurology faculty with >4 years' experience as teaching faculty. The medical educator was a full-time education specialist with >4 years' experience in the undergraduate medical education setting. When providing narrative feedback, experts had access to the key points for each case.

**Figure 2:**
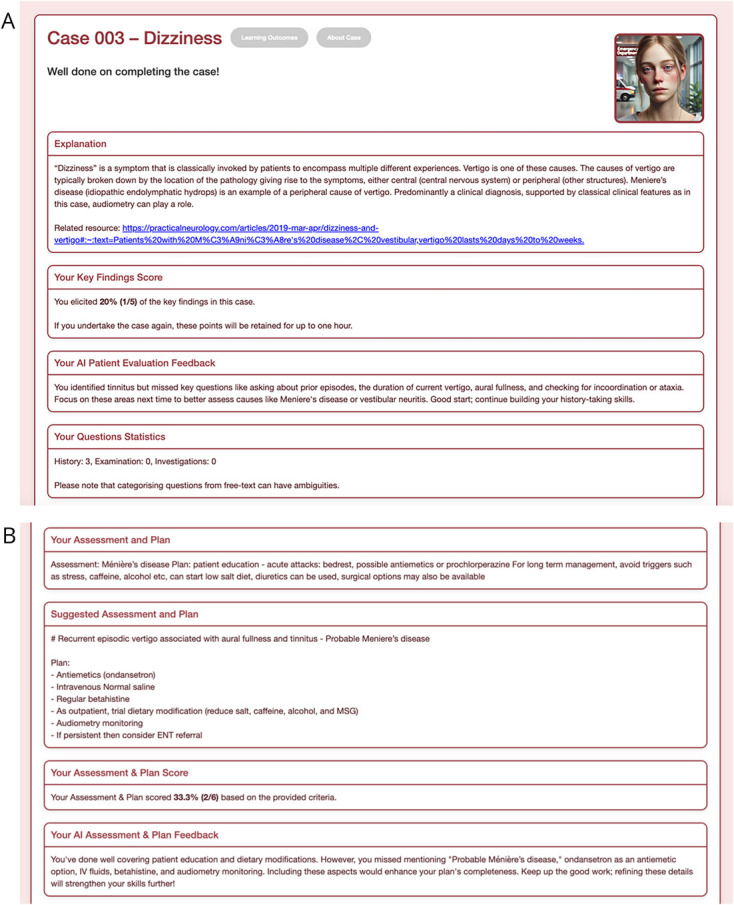
TEACHABLE Case Feedback (A) Sample output feedback provided by the large language model (LLM) to learners on their history, examination, and investigations based on key findings established by expert writers for the case. (B) Sample output feedback provided by the LLM to learners on their assessment and plan based on key findings established by expert writers for the case.

An LLM was also provided with student transcripts and asked to provide feedback (Figure 2, A and B). The prompt provided to the LLM asked it to review the student transcripts against the key learning points of each case. The prompt also included a summary that characterized how often students had asked open history questions, closed history questions, examination findings, and investigation results. Human experts had access to the raw questions asked by trainees, so they could evaluate how often questions were open ended or closed ended, how many pertained to history, examination elements, etc. Although the LLM had access to the same information as the human assessors, these data were summarized for the LLM to make the feedback focused and specific. This prompt engineering was used to improve the quality and consistency of the feedback (eAppendix 3 includes an annotated version of the prompt). The LLM generated narrative feedback for the history-taking domains. The LLM used for this task was *OpenAI's* GPT-4o.^21^

Neither the human experts nor the LLM were explicitly told how the quality of their feedback would be evaluated or directed to follow a specific rubric or approach to feedback. This method was used to assess the default method of feedback generation used by both the human experts and the LLM and to mimic a real-world setting. However, both were prompted to focus feedback on the case's key learning points to ensure alignment of feedback and CBL objectives.

#### Part 2: Assessment and Plan Evaluation

The same methods as above were used to generate feedback by the experts and the LLM on student performance for the A&P formulation; the only difference was that 2 clinicians provided feedback on the assessment/plan domains because they possessed the expertise for clinical reasoning (one was a neurologist with >2 years' experience and the second a hematologist with >13 years' experience). The LLM was promoted to provide feedback on this task as above. LLM feedback was available immediately after prompting.

### Feedback Comparison

#### Feedback Characteristics

LLM-generated and human-generated feedback were described through word and sentence counts. Manual review of all feedback was undertaken by one medical education consultant; each individual item of feedback was categorized dichotomously as to whether it commented specifically on the key learning points of the case or not. Similarly, each feedback item was classified dichotomously as to whether it commented on each of the components of the diagnostic evaluation (i.e., history, examination, and investigation content) or not.

#### Feedback Quality

For both parts 1 and 2, the quality of the feedback generated was evaluated by 2 independent, blinded expert evaluators (2 experts for each part, 4 experts in total). The expert evaluators were neurology educators with a range of 2–15 years of experience. All held education leadership roles in which they had experience providing feedback and reviewing feedback from other teaching faculty. The feedback generated by human experts and the LLM was provided to the evaluators and scored using 2 previously published scores for evaluating narrative feedback quality in education and workplace settings: the Quality of Assessment for Learning (QuAL) scale and the EFeCT Feedback Scoring Tool (EFeCT).^18,19^ The QuAL and EFeCT instruments were selected because both have validity evidence supporting their use as standardized measures of written feedback quality in medical education.^19,22^ The QuAL and EFeCT scores range from zero to 5 and assess areas such as provided context, feedback specificity, and perceived utility for students, providing a quantitative indicator of feedback quality. The blinded raters familiarized themselves with these instruments and used a standardized grading rubric with the components of both instruments referenced explicitly.

Thus, each piece of feedback received a total of 2 quality scores. Finally, as a safeguard, the LLM feedback was reviewed for medical accuracy by a medical education consultant.

### Data Analysis

Descriptive statistics were used to present feedback item characteristics, including word length, sentence length, dichotomized discussion of learning points (present/absent), and dichotomized inclusion of all content components (present/absent). Feedback characteristics (word counts and sentences counts) were found to be not normally distributed, thus nonparametric testing with the two-sample Wilcoxon rank-sum (Mann-Whitney) test was conducted to compare human expert‑generated and LLM-generated feedback groups.

The overall quality evaluations for human expert‑generated vs LLM-generated feedback were calculated as median scores for QuAL and EFeCT and compared. For H&P, expert performance was averaged at the level of individual cases (n = 5) and students (n = 4), yielding 20 LLM and 60 expert feedback items (from 3 experts) for comparison. For A&P, expert performance was averaged at the level of individual cases (n = 5) and students (n = 4), yielding 20 LLM and 40 expert feedback items (from 2 experts) for comparison.

The total QuAL and EFeCT scores of the feedback as determined by 2 evaluators were averaged to generate a single feedback quality score per case for each student. This generated a total of 20 average human expert and 20 LLM feedback quality scores to compare. The QuAL and EFeCT scores are reported as continuous numerical variables in their original publications.^18,19^ However, because our results were not normally distributed, we reported median and used nonparametric tests of significance. The two-sample paired Wilcoxon signed-rank test was conducted to compare median scores from human expert‑generated and LLM-generated feedback groups. The significance value was set at *p* < 0.05 for all comparisons. Statistical analysis was performed using Excel (version 16.78, Microsoft, WA) and Python (version 3.14.3, Python Software Foundation, OR).

### Ethical Approval

This pilot study used a publicly available LLM and student and faculty coinvestigators whose data were deidentified and anonymized; therefore, formal ethics approval was not required.

### Data Availability

Anonymized data not published within this article will be made available by request from any qualified investigator.

## Results

### Feedback Characteristics

#### Part 1: History Taking/Physical Examination Elements Feedback

For feedback related to H&P, the median number of words in the LLM feedback was 47 (IQR 44.5 to 50.0), as compared with 43 (IQR 16.0 to 103.5) for the human expert feedback (Z = −0.38, *p* = 0.71). Similarly, the number of sentences per item of LLM feedback was 4 (IQR 3 to 4) as compared with 3 (IQR 2 to 6) for the experts (Z = −0.88, *p* = 0.38). The LLM commented on aspects of the key learning points in 20/20 (100%) items of feedback, compared with 39/60 (65%) for the human experts. The number of instances in which the LLM feedback included discussion of the history, examination, and investigations was 20/20 (100%), 18/20 (90%), and 11/20 (55%) as compared with experts with 60/60 (100%), 50/60 (83.3%), and 34/60 (56.7%), respectively.

#### Part Two: Assessment and Plan Feedback

When characterizing feedback on the A&P component of the transcripts, the median number of words in the LLM feedback was 49.5 (IQR 47.5 to 52.0), as compared with a median of 74.5 (IQR 57.75 to 99) for the expert feedback (Z = 4.36, *p* < 0.0001). The median number of sentences in the LLM feedback was 4 (IQR 4 to 5) and the median for the expert feedback was also 4 (IQR 3 to 5.5) (Z = −0.05, *p* = 0.96). The LLM feedback discussed key points relating to the scenario in 20/20 (100%) cases, and the human-generated feedback discussed key points in 39/40 (97.5%) cases. Furthermore, the LLM feedback mentioned differential diagnoses in 6/20 (30%) cases, additional testing in 15/20 (75%) cases, and treatment options in 20/20 (100%) cases. In the human-generated feedback, these rates were 13/40 (32.5%), 30/40 (75%), and 38/40 (95%), respectively.

### Feedback Quality

#### Part 1: History Taking/Physical Examination Elements Feedback

The blinded expert evaluators gave the LLM feedback pertaining to H&P a higher median QuAL score of 5.0 (IQR 5.0–5.0), as compared with the median human expert feedback score of 4.7 (IQR 4.29–4.67) (W = 0.0, *p* < 0.00001, r = 1.0). For the EFeCT score, the expert evaluators gave the LLM feedback a higher median score of 5.0 (IQR 5.0–5.0) compared with an average median score of 4.7 (IQR 4.29–4.67) (W = 0.0, *p* < 0.0001, r = 1.0) for human expert feedback. All 20/20 (100%) of the LLM feedback items were medically accurate. We examined which components of the scales drove differences between expert-generated and LLM-generated feedback. The LLM feedback was consistent and received scores of 5/5 on each case from both blinded reviewers for both QuAL and EFeCT instruments. Human feedback was less consistent and received variable scores. For the QuAL, human feedback lost points in the question, “Does the assessor provide sufficient evidence about learner performance?” This is graded as 0 = no comment at all; 1 = no, but comment present; 2 = somewhat; and 3 = yes, full description. While the LLM always scored 3, human feedback was rated 1, 2, or 3. For the EFeCT, the largest difference between human-generated and LLM-generated feedback was for the “Context” component where feedback had to contain “When, who, where—some cue about the type of patient and their demographics or context/symptoms).”

#### Part Two: Assessment and Plan Feedback

When evaluating the quality of A&P-related feedback, no differences were found between average expert ratings of the LLM-generated feedback and the human expert‑generated feedback for either measure. The LLM median QuAL score was 5.0 (IQR 5.0–5.0) compared with the median QuAL score for human feedback of 5.0 (IQR 5.0–5.0) (W = 4.0, *p* = 0.34, r = 0.47). The median EFeCT score for LLM feedback was 4.5 (IQR 4.5–4.5) compared with the median EFeCT score for human feedback of 4.3 (IQR 4.25–4.56) (W = 43.0, *p* = 0.53, r = 0.18). All 20/20 (100%) of the LLM items of feedback were evaluated as medically accurate.

## Discussion

This pilot study demonstrates that an LLM provided with a rubric of key points can generate narrative feedback on medical students' case-based interactions that is comparable in content and quality to feedback provided by human experts. Quantitative evaluation by blinded expert raters using established metrics for feedback quality (using the QuAL and EFeCT scores) revealed that LLM-generated feedback received significantly higher ratings than human expert‑generated feedback for the H&P component and no significant difference was noted for the A&P component. No medical inaccuracies were identified in the LLM outputs. This suggests an LLM can produce accurate feedback reliably and rapidly that is at least comparable with human experts in quality.

High-quality feedback in medical education should be timely, reliable, specific, and actionable.^11,13,14^ Constructive, real-time feedback helps learners identify gaps, solidify correct reasoning patterns, and apply improvements to future encounters. In a study of bedside examination teaching, for example, structured, feedback-driven near-peer teaching enhanced residents' confidence and engagement.^15^ Thus, when comparing the output of LLM-generated feedback with human expert‑generated feedback, we wanted to ensure it was not only delivered rapidly but was also accurate, specific, reliable, and actionable. First, no medical inaccuracies were found in the LLM feedback. Second, LLM-generated feedback showed greater consistency in length across students and cases compared with faculty feedback. We did prompt the LLM to provide feedback within a limited word count (see eAppendix 3). This demonstrates LLM-generated feedback can be intentionally optimized through thoughtful prompt engineering. Third, the content was reliable and specific, aligned with case key points. The LLM demonstrated high consistency in addressing all key points of the students' H&P and A&P in its feedback. Quantitative analyses showed comparable inclusion of key H&P elements, differential diagnoses, additional testing, and treatment considerations when compared with expert-generated feedback. By reinforcing what students did well and identifying gaps for improvement, LLM feedback may help build students' reasoning through deliberate practice.

Prior research has established that LLMs can support higher order reasoning tasks by scaffolding clinical decision-making processes.^4,23^ Brügge and colleagues^17^ showed that LLM-generated feedback for CBL improves clinical decision-making skills among medical students using a static, traditional format. Brugge et al. reported on final student outcomes and found LLM feedback improved learner performance; however, they did not describe the quality of the feedback provided or use measures to compare the quality of LLM vs faculty feedback. Future research should continue to explore the role of feedback in augmenting these processes.

In addition to comparing feedback content, we assessed the quality of the feedback with 2 blinded scorers who used the QuAL and EFeCT scores, 2 formal instruments for evaluating quality of feedback with prior validity evidence for use in medical education settings. We found the quality of LLM-generated feedback was at par with human expert‑generated feedback for the A&P component and of higher quality for the H&P component. The LLM feedback was consistently rated as receiving a perfect median score on the QuAL and EfeCT instruments. We posit that the LLM outperformed human experts for the H&P component given the highly structured nature of this task. Eliciting the history and examination involves a structured, checklist-based approach, which may be easier for the LLM to identify and consistently rate across students as compared with human experts. In addition, human experts may have sought to selectively prioritize specific elements of the student response to give feedback on which they deem more urgent or imperative to shape future performance of the learner, while the LLM may have more consistently and systematically identified each discrepancy from the provided checklist. The benefits of each method and the right balance between providing comprehensive feedback and choosing specific areas to focus on so as not to overload the learner is an important area for future study.

The LLM-generated feedback was at par with faculty-generated feedback for the A&P component, but not of higher quality. The A&P task requires higher order clinical reasoning and synthesis of diagnostic and management information by learners. This is a task with higher complexity and a larger set of possible accurate answers, which depend on justification. Generating an A&P is often more cognitively challenging than history gathering alone. It is noteworthy therefore that the performance of the LLM in providing feedback on students' A&P formulation was at least at par with expert faculty despite increased task complexity. Overall, the LLM maintained medical accuracy while offering timely, structured guidance for improvement—qualities that may be particularly valuable in formative contexts where learners are still developing their clinical diagnostic frameworks and require ongoing practice.

As medical education programs grow, with increasing student-to-faculty ratios, there is a rising emphasis on identifying innovative initiatives that can be delivered consistently at scale, to ensure an equitable learning environment and opportunities for all students. In our study, the LLM-generated feedback showed greater reliability in feedback length and better consistency in addressing specific key points prioritized in the case-specific learning objectives. Our results suggest that LLMs may not only serve as a supplement to expert feedback but could also standardize the delivery of feedback, mitigating the variability that can be seen in the depth and focus of feedback delivered by several human reviewers.^12,17^ LLMs can also consistently apply the same evaluative framework across all submissions without being subject to fatigue, cognitive bias, lack of faculty, or differences in interpretive standards.^16,24^ This consistency can enhance the reliability of formative assessment, particularly in large-scale courses and multisite programs. Incorporating LLM feedback could help standardize formative assessment of clinical reasoning at scale, supporting learners in refining diagnostic reasoning and critical reflection.^24^

Immediate and individualized feedback across large cohorts of learners can further support high-quality self-directed learning. Immediate AI feedback may enable learners to adapt rapid cycles of revision and self-correction, encouraging reflective practice and enhancing self-improvement. By allowing students to independently engage in cycles of case-based reasoning with immediate, structured feedback, LLMs may help approximate the conditions of deliberate practice more reliably than the clinical environment alone, where not all learner actions and decisions are observed, and feedback may not be specific or delivered in a timely manner. We did not formally record the time taken by faculty to provide feedback. However, faculty time for feedback is estimated to be in the range of hours. Future work could look objectively at the time and resources required for human vs AI-derived feedback to inform real-world application. Finally, the accuracy of LLM feedback may be further enhanced by having instructional designers create specific rubrics or frameworks tailored to the activity a learner is engaging in. In this way, LLM-generated feedback does not replace human teaching but rather enhances the educational value of AI-enabled CBL by making deliberate practice accessible at scale.

Our work expands upon prior research using LLMs to generate feedback in nonclinical learning environments. For example, LLMs can produce useful feedback for multiple-choice questions^23^ and secondary school writing classes, supporting student motivation and guiding revisions.^25^ A recent multi-institutional study demonstrated students perceive AI feedback to be more accessible and understandable, while teacher feedback was viewed as more helpful and trustworthy; the authors emphasized that these modalities are not duplicative but may be complementary.^26^ In future investigations, acceptability and perceptions of LLM feedback among medical students should be explored, recognizing that learner acceptance of feedback influences meta-cognition and self-directed learning and improvement.^27^ The effect of delivering high-quality, timely LLM feedback on learner performance should also be a priority for future study. Given that LLM feedback more reliably addressed key learning points compared with human expert feedback in our study, further research could explore whether this reliability improves acquisition of clinical reasoning over time or influences behavior to improve students' subsequent clinical performance.

The implementation of LLM-generated feedback for students' self-directed learning offers new avenues of possibility for educators. Reviewing aggregated LLM feedback may reveal trends in learner misconceptions and common errors, helping educators identify gaps to prioritize in curricular quality improvement. Incorporating LLM feedback alongside expert oversight in a hybrid format could optimize learning efficiency, standardize evaluations, and foster equitable access to high-quality formative assessment.^28^ Embedding LLM systems within institutional learning platforms could enable automated tracking of student engagement and progress through dashboards and audit trails. Such integration allows educators to monitor how students interact with feedback, identify learning trends, and track when intervention with supervision is needed.

With the rapid pace of LLM advancement, the capabilities of these systems to support education will continue to evolve.^23^ Making the most of these possibilities will require coordinated collaboration among educators, learners, academic institutions, researchers, and technology developers.^29^ Institutions will need to establish appropriate guardrails to ensure privacy of student data, equitable access, and ongoing faculty oversight. Moreover, faculty development in prompt design and interpretation of LLM-generated output, and periodic critical review of AI integration will be essential to ensure quality assurance, consistency, and alignment with curricular goals.

This pilot feasibility study is limited by the small number of student participants and expert reviewers. We focused our pilot on subspeciality cases in neurology; generalizability may be limited beyond this domain. However, given feedback quality was assessed as compared with key learning points, we imagine this could be replicated for cases in other medical specialties. All analyses were conducted in English, although the study took place in multiple geographic sites (the United States and Australia). A strength was the use of established quality scoring instruments and blinded scorers. This was a feasibility study that took place in an artificial/simulated setting, and thus the performance of the human experts in this study may not fully represent the practice of students or educators functioning within real-world contexts. Expanded work should evaluate implementation of LLM-generated feedback in situ within educational programs with diverse cohorts of learners and experts. Feedback generated by LLMs is also highly contingent upon effective prompting; this warrants systematic evaluation to ensure reproducibility across various programs, LLM versions, and prompts. Optimal prompting may need to be further evaluated and replicated. We acknowledge LLMs are routinely updated, so subsequent versions of OpenAI's GPT may yield different results in the future. We did not evaluate if elements of the language, syntax, or style of feedback may have cued blinded raters into thinking the feedback was generated by a LLM or a human and did not do a postassessment survey to ask reviewers about this. This would be a valuable addition in future studies comparing human and LLM outputs.

In a pilot feasibility study, we showed a LLM provided with a rubric of key points can give feedback to learners on case-based interactions in neurology in a manner that is timely, reliable, and of comparable quality to feedback generated by human experts. Our results are contingent on effective prompt design, and their larger scale impact may depend on LLM integration with existing instructional systems. Nonetheless, our findings demonstrate the promise of leveraging LLMs to provide students with high-quality, timely, and specific feedback, which in turn may enhance opportunities for deliberate practice during self-directed case-based learning.

## Glossary

AI: artificial intelligence
CBL: case-based learning
LLM: large language model
QuAL: Quality of Assessment for Learning
